# MnSOD and Cyclin B1 Coordinate a Mito-Checkpoint during Cell Cycle Response to Oxidative Stress

**DOI:** 10.3390/antiox6040092

**Published:** 2017-11-17

**Authors:** Amanda L. Kalen, Iman M. Ahmad, Maher Y. Abdalla, Yunxia Q. O’Malley, Prabhat C. Goswami, Ehab H. Sarsour

**Affiliations:** 1 Free Radical and Radiation Biology Program, Department of Radiation Oncology, University of Iowa, Iowa City, IA 52242, USA; amanda-kalen@uiowa.edu (A.L.K.); prabhat-goswami@uiowa.edu (P.C.G.); 2 Department of Medical Imaging and Therapeutic Sciences, College of Allied Health Professions, University of Nebraska Medical Center, Omaha, NE 68198, USA; Iman.ahmad@unmc.edu; 3 Department of Pathology and Microbiology, College of Medicine, University of Nebraska Medical Center, Omaha, NE 68198, USA; maher.abdalla@unmc.edu; 4 Department of Pediatrics, University of Iowa, Iowa City, IA 52242, USA; yunxia-omalley@uiowa.edu

**Keywords:** cyclin B1, MnSOD, cell cycle, oxidative stress mitochondria, reactive oxygen species

## Abstract

Communication between the nucleus and mitochondrion could coordinate many cellular processes. While the mechanisms regulating this communication are not completely understood, we hypothesize that cell cycle checkpoint proteins coordinate the cross-talk between nuclear and mitochondrial functions following oxidative stress. Human normal skin fibroblasts, representative of the G_2_-phase, were irradiated with 6 Gy of ionizing radiation and assayed for cyclin B1 translocation, mitochondrial function, reactive oxygen species (ROS) levels, and cytotoxicity. In un-irradiated controls, cyclin B1 was found primarily in the nucleus of G_2_-cells. However, following irradiation, cyclin B1 was excluded from the nucleus and translocated to the cytoplasm and mitochondria. These observations were confirmed further by performing transmission electron microscopy and cell fractionation assays. Cyclin B1 was absent in mitochondria isolated from un-irradiated G_2_-cells and present in irradiated G_2_-cells. Radiation-induced translocation of cyclin B1 from the nucleus to the mitochondrion preceded changes in the activities of mitochondrial proteins, that included decreases in the activities of aconitase and the mitochondrial antioxidant enzyme, manganese superoxide dismutase (MnSOD), and increases in complex II activity. Changes in the activities of mito-proteins were followed by an increase in dihydroethidium (DHE) oxidation (indicative of increased superoxide levels) and loss of the mitochondrial membrane potential, events that preceded the restart of the stalled cell cycle and subsequently the loss in cell viability. Comparable results were also observed in un-irradiated control cells overexpressing mitochondria-targeted cyclin B1. These results indicate that MnSOD and cyclin B1 coordinate a cross-talk between nuclear and mitochondrial functions, to regulate a mito-checkpoint during the cell cycle response to oxidative stress.

## 1. Introduction

The main function of cell cycle regulatory proteins is to drive cell progression from one phase of the cell cycle to the next and invoke a cell cycle checkpoint during conditions of stress. This is primarily a nuclear event and involves DNA damage, which allows the cells time to undergo repair process; other cell organelles could also be affected by this process. Cyclins are major regulators of cell cycle progression: cyclin D1 drives the cells from G_1_ to S, cyclin E regulates cell S phase progression, cyclin A transits cells into G_2_ from S, and cyclin B1 drives cells progression through G_2_ and into the M phase of the cell cycle. However, recently these cell cycle regulatory proteins were proposed to have functions beyond their primary role of regulating the cell cycle.

In several studies, cyclin D1 has been found to not only regulate transition from the G_1_ to the S phase of the cell cycle, but also it acts as a regulator of mitochondrial functions [1,2,3]. One study demonstrated that cyclin D1 inhibits mitochondrial activity and aerobic glycolysis in vivo [2]; another study showed that cyclin D1 coordinates nuclear DNA synthesis and mitochondrial function [3]. A recent study also showed that cyclin B1 improves mitochondrial function during adaptive cellular responses to radiation [4]. In studies of our own, we have shown that mitochondrial functions and mitochondrial protein activity could affect nuclear functions and proteins [5,6,7,8]. We have shown a balance between two critical nuclear proteins—p16 and p21—could mediate mitochondrial antioxidant enzyme, manganese superoxide dismutase (MnSOD) induced protection of the proliferation capacity of the quiescent fibroblasts [7]; in another study we showed that cross-talk between MnSOD activity (possibly *via* mitochondria derived reactive oxygen species (ROS) levels) and cell cycle regulatory proteins, cyclin D1 and B1 could significantly influence the transitions between quiescent and proliferative growth [8].

These studies strongly suggest that communication between cellular compartments during the cell cycle is essential for cellular functions and cell cycle regulation, but the effects of such communications on cell cycle progression and response to oxidative stress are still not well understood. To investigate such communication, we used bioinformatic evaluation of cyclin protein sequences for mitochondrial translocation signals. We used MitoProt II 1.0a4 software (Institut für Humangenetik Technische Universität, München, Germany) to search for mitochondrial localization signals. The software identified a twenty-two N-terminal amino-acid sequence motif in cyclin B1 protein (MALRVTRNSKINAENKAKIN, 10 basic and 1 acidic residue) as the mitochondria target sequence, with a probability score of 0.81 for mitochondria localization [9]. Cyclin B1 is known to translocate from the cytoplasm to the nucleus during cell cycle progression through G_2_, and is excluded from the nucleus to the cytoplasm during the activation of the G_2_ checkpoint and cell cycle delay.

In this study we examined the translocation of cyclin B1 during the G_2_ checkpoint activation in response to oxidative stress and its effects on mitochondrial function, with respect to cell cycle progression and cell viability.

## 2. Material and Methods

### 2.1. Cell Line and Culture Conditions

Human normal skin fibroblasts (NHF, AG01522) were obtained from Coriell Cell Repositories, and human epithelial mammary gland cells (MCF10A, ATCC CRL-10317) were maintained in Dulbecco’s modified Eagle’s medium (DMEM high glucose, Invitrogen) with 10% fetal bovine serum (FBS, HyClone Laboratories, South Logan, UT, USA), supplemented with penicillin/streptomycin antibiotics (1:500 final concentration, prepared with 10,000 unit/mL penicillin G sodium and 10,000 µg/mL streptomycin sulfate in 0.85% saline, Invitrogen). Cells were grown in incubators with a controlled temperature of 37 °C, CO_2_ concentration of 5%, humidity of 95% and 21% O_2_. Cells were harvested either by scraping or by trypsinization with 1× trypsin (trypsin 0.05% EDTA in phosphate buffered saline (PBS), Invitrogen). The University of Iowa Radiation and Free Radical core facility, housing a cesium 137 source, was used to irradiate cells; the dose rate was 0.89 Gy per min.

### 2.2. Cell Viability Assay

A flow cytometry assay of propidium iodide (PI) incorporation into non-fixed cells was used to measure the percentage of non-viable cells. Cells were trypsinized, harvested, and kept on ice. After centrifugation, the pellets were re-suspended in ice cold PBS. PI was added at a final concentration of 1 μg/mL and incubated for 5 min on ice prior to analysis. The percentages of PI positive (non-viable) and PI negative (viable) cells were calculated using FlowJo software (FlowJo LLC, Ashland, OR, USA).

### 2.3. Flow Cytometry Assay of DNA Content

Ethanol-fixed cells were treated with RNase A for 30 min, followed by staining with PI. PI-stained cells were analyzed by flow cytometry and 10,000 events were collected in list mode. Cell cycle phase distributions were calculated using ModFit software (Verity Software House, Topsham, ME, USA).

### 2.4. Sub-Cellular Fractionation

Cell fractionation and mitochondrial isolation were performed following the manufacturer supplied protocol using Qproteome Mitochondria Isolation Kit (Qiagen, Germantown, MD, USA), a cell fractionation kit for mitochondrial purification.

### 2.5. Immunoblotting Assay

Cells were harvested by either scraping or trypsinization, washed with PBS, and re-suspended in phosphate buffer (pH 7.8). Protease and phosphatase inhibitors were added to the preparations of all protein extracts (Protease inhibitors cocktail, Sigma and Phospho-Stop, Roche). Samples were sonicated on ice, with 5 bursts of 15 s, using a Vibra Cell Sonicator with cup attachment (Sonics and Materials Inc., Newtown, CT, USA). Protein concentrations were determined with a Bradford protein assay kit (Bio-Rad Laboratories, Hercules, CA, USA). Equal amounts of proteins were separated by 12.5% SDS-PAGE, electro-transferred by semidry blotting onto a nitrocellulose membrane, and probed with antibodies to MnSOD, cyclin B1, and Complex I. Immunoreactive bands were detected by an enhanced chemiluminescence kit. The bands were visualized using X-ray film and imaged with computerized digital imaging system using AlphaImager 2000 software (Alpha Innotech, San Leandro, CA, USA). Bands were quantified with ImageJ software (ImageJ, National Institute of Health, Bethesda, MD, USA). The integrated density value was obtained by integrating the entire pixel values in the area of one band after correction for background. Actin protein levels were used for loading corrections.

### 2.6. MnSOD Activity Gel Assay

Total cellular protein extracts were assayed for MnSOD activity. Equal amounts of proteins were run through 12.5% non-dissociating (native) gel and a 5% stacking gel containing riboflavin. Gels were polymerized under white fluorescent light. After electrophoresis, gels were stained for superoxide dismutase (SOD) activity using nitroblue tetrazolium (NBT) (2.43 mM) and riboflavin-TEMED (riboflavin 2.8 × 10^−5^ M and TEMED 28 mM) for 20 min at room temperature. Gels were kept in distilled water and illuminated under bright fluorescent light until SOD bands were visible (1–4 h). Sodium cyanide (0.75 mM) was added to distinguish between CuZnSOD and MnSOD activities. The bands were visualized and quantified with a computerized digital imaging system interfaced with AlphaImager 2000 software (Alpha Innotech).

### 2.7. Complex I, II, IV and Aconitase Activity Assays

Aconitase activity was determined by following a previously published protocol [10]. Briefly, 20 µg of mitochondria protein extract was mixed with a reaction buffer (50 mM Tris pH = 7.4, 1 U/mL isocitrate dehydrogenase, 0.6 mM MnCl_2_, 400 µM cis-aconitate, and 200 µM NADP), and the optical density (OD) of this mixture was measured at 340 nm, 37 °C for 15 min. Previously published protocols were used to assay for complex I, II, and IV activity [11]. Briefly, 20 µg of mitochondria protein extract was mixed with a reaction buffer containing the following: for complex I (25 mM potassium phosphate pH = 7.2, 5 mM MgCl_2_, 2 mM KCN, 100 µM NADH, 65 µM ubiquinone, 1 mg/mL antimycin A and 1 mg/mL rotenone), for complex II (25 mM potassium phosphate pH = 7.2, 5 mM MgCl_2_, 2 mM KCN, 20 mM sodium succinate, 65 µM ubiquinone, 1 mg/mL antimycin A and 1 mg/mL rotenone, and 100 µM 2,6-dichlorophenolindophenol), for complex IV (25 mM potassium phosphate pH = 7.2, 30 mM Dodecyl-maltoside and 50 µM Cytochrome c (II)). Complex I activity was measured by following the decrease in the absorbance due to the oxidation of the nicotinamide adenine dinucleotide (NADH) at 340 nm for 10 min at 37 °C; complex II activity was measured by following the decrease in the absorbance due to the reduction of dichlorophenolindophenol at 590 nm for 10 min at 37 °C; and complex IV activity was measured by following the increase in the absorbance due to the oxidation of cytochrome c (II) at 540 nm for 10 min at 37 °C.

### 2.8. Immune-Staining and Confocal Microscopy

Monolayer cultures of control and treated cells, grown in chamber slides, were fixed in 4% para-formaldehyde, washed, and incubated with 0.2% Triton X-100. Cells were incubated with 5% goat serum for 30 min and incubated with a mixture of primary antibodies of rabbit anti-human MnSOD and mouse anti-human cyclin B1, followed by incubation with a mixture of secondary antibodies—goat anti-rabbit Alexa 546 (red) and goat anti-Mouse Alexa 488 (green). Cells were counter stained with TO-PRO3 for nuclear staining (blue). A Zeiss 510 confocal microscope was used for visualization and data storage (The University of Iowa Central Microscopy Research Core Facility). Co-localization was evaluated with ImageJ software, to calculate the co-localization coefficient, using Mander’s Overlap coefficient [9] method, where the intensity of a given pixel in the first channel image is used as one coordinate to the intensity of the corresponding pixel in the second channel; then, the intensity of each pixel is correlated between the two images for the (red/green) values, where values closer to 1 are considered correlated and co-localized. Red-green correlation plots were generated using the same software.

### 2.9. Immuno-Gold Staining and Electron Microscopy

Electron microscopy was performed following our previously published protocol [7]. Briefly, monolayer cells were collected by scraping and fixed with a light fixative mixture of 4% para-formaldehyde and 0.05% glutaraldehyde. Fixed cells were then centrifuged and pellets were washed three times with 0.1 M Na-cacodylate buffer, followed by incubation with 1% OsO_4_ for 2 h, and then were processed for embedding by incubating with 2.5% uranyl acetate (20 min). Ultra-thin sections (70 nm) were incubated with mouse anti-human cyclin B1 antibodies and F(ab)2 goat anti-mouse ultra-small gold-conjugated secondary antibodies. Samples were visualized using JEOL 1230 transmission electron microscopy at the University of Iowa Central Microscopy Research Facility.

### 2.10. Mitochondria-Targeted Expression of Human Cyclin B1

High fidelity PCR amplification was performed to amplify the 1.5 kb human cyclin B1 cDNA, cloned into pGEM plasmid DNA (gift from Dr. Ruth Muschel, University of Pennsylvania). Sal 1 and Not 1 restriction enzyme sites were added to the forward and reverse primers, respectively, for directional cloning; the forward primer was: 5′ TTG TCG ACG CGC TCC GAG TCA CCA GG AAC 3′ and the reverse primer was 5′ AAT GCG GCC GCC ACC TTT GCC ACA GCC TTG 3′. PCR-amplified DNA was purified by gel electrophoresis, extracted, and cloned in-frame to the Sal I and Not I sites of the pShooter vector (pCMV/myc/mito, Invitrogen). The pShooter vector incorporated a mitochondrial localization signal sequence at the N-terminal and a myc-epitope at the C-terminal. The inserted sequence was verified by sequencing (The University of Iowa DNA/Vector Core Facility). Cells were transfected with control and cyclin B1 cDNA containing plasmids. Immunostaining and confocal microscopy were used to verify transgene expression and localization of cyclin B1 protein.

### 2.11. Dihydroethidium (DHE) for ROS Levels and Mitochondrial Membrane Potential Assays

Cultures were incubated with Hanks buffer salt solution (HBSS) containing 10 µM DHE (Invitrogen, USA) for 45 min. Cultures were trypsinized and re-suspended in HBSS buffer containing 10% FBS. The flow cytometry measurements of DHE oxidation were performed using 488 nm excitation laser and FL2 channel- 585/42 nm band pass emission filter. Mean fluorescence intensity (MFI) was analyzed using Flowjo software. Auto-fluorescence of unlabeled cells was used for background fluorescence correction. For mitochondrial membrane potential, we used a JC-1 assay. Briefly, cells were washed with HBSS. The staining solution was prepared by diluting the JC-1 stock solution (Invitrogen, Carlsbad, CA, USA) with HBSS (final concentration, 2 µg/mL). Two mL of the staining solution was applied to cells and incubated for 10 min at 37 °C. Cells were then collected by trypsinization, washed, filtered and analyzed immediately with flow cytometry equipped with a 488 nm argon laser. The value of photomultiplier (PMT) detecting the signal in FL1 was set at 390 V, and FL2 PMT at 320 V; FL1-FL2 compensation was around 4.0%, while FL2-FL1 compensation was around 10.6%. Ratios between FL1 to FL2 were used to calculate the percent loss of mitochondrial membrane potential compared to untreated controls. CCCP membrane uncoupler was added to cells as a positive control.

## 3. Results

### 3.1. Cyclin B1 Translocates to Mitochondria Following Oxidative Stress Affecting Mitochondrial Function

**Figure 1:**
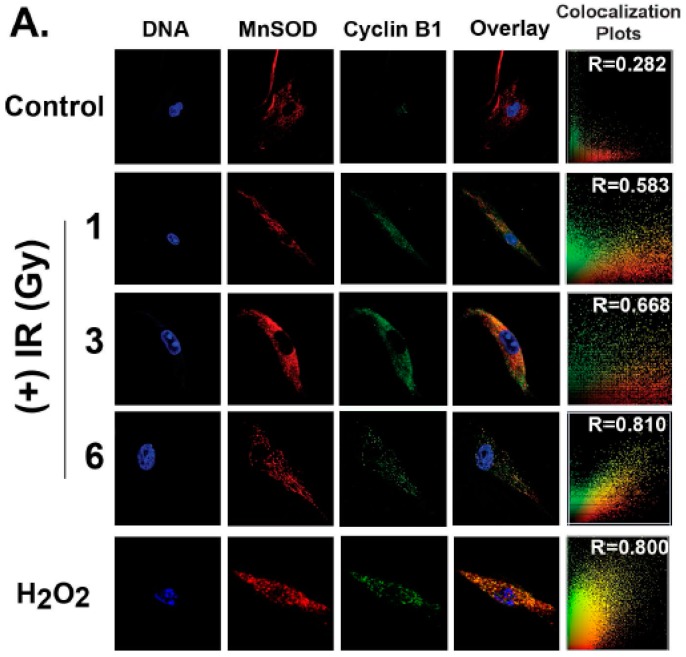

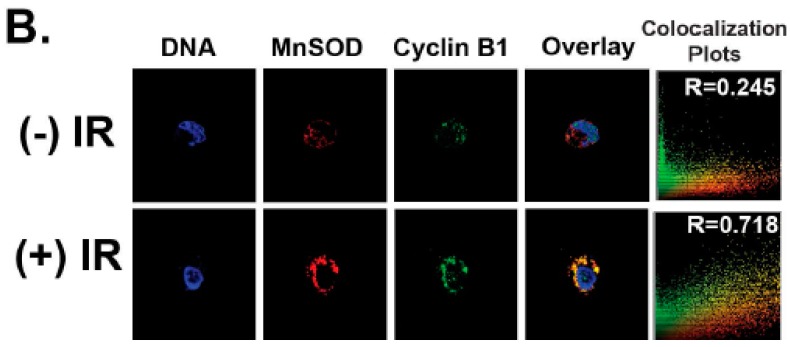
Cyclin B1 mitochondria translocation. (**A**) Representative images of confluent normal human skin fibroblasts (NHFs): NHFs were re-plated and irradiated at 18 h post-plating (representative of late S and G_2_ phases) with 1–6 Gy (dose rate: 0.89 Gy/min) ionizing radiation (IR). At 4 h post-irradiation, monolayer cultures were fixed and processed for immunostaining and confocal microscopy; TO-PRO3 (nuclear, blue staining), MnSOD (mitochondria, red staining), cyclin B1 (green staining), and overlay images of MnSOD and cyclin B1; co-localization plots show red and green overlapping distributions and calculated Mander’s Overlap coefficient (R). When the red/green ratio closer to 1, they are considered correlated and co-localized. The bottom panel represents NHFs treated with 250 µM hydrogen peroxide for 1 h. (**B**) Representative images of exponentially growing asynchronous cultures of human non-malignant mammary epithelial cells (MCF10A), irradiated with 6 Gy and harvested at 4 h post-irradiation for immunostaining and microscopy assay.

To determine if oxidative stress activates nuclear to mitochondria cross-talk, human normal skin fibroblasts (NHFs) were irradiated with 1–6 Gy and harvested 4 h post-irradiation for immunostaining and microscopy assays. Cyclin B1 was found primarily in the cytoplasm of 1, 3, and 6 Gy irradiated NHFs (Figure 1A). Immunostaining with MnSOD showed cyclin B1 translocated to the mitochondrion in irradiated cells, detected as punctate yellow staining (overlapping of green (cyclin B1) and red (MnSOD) staining). The percentage of cells that had cyclin B1 translocating to mitochondria corresponded to the percentage of G_2_ cells after the radiation treatment (Figure 5). Cyclin B1 mitochondria translocation showed a dose-dependent increase, with a maximum co-localization coefficient of 0.81, in 6 Gy irradiated cells. Cyclin B1 mitochondria localization in hydrogen peroxide treated NHFs further suggested that oxidative stress could translocate cyclin B1 to the mitochondrion. Cyclin B1 mitochondria translocation in MCF10A human mammary epithelial cells (Figure 1B) indicated cell type independence of the phenomenon.

**Figure 2:**
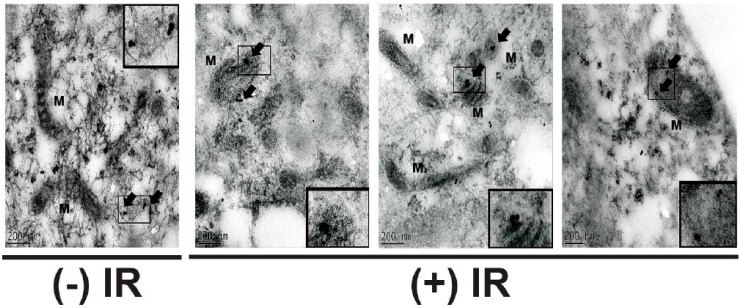
Immunogold staining and transmission electron microscopy demonstrating cyclin B1 mitochondria translocation. The control and 6 Gy irradiated NHFs were harvested at 4 h post-irradiation for the electron microscopy assay, following our previously published protocol (11). The box shows sections of the mitochondrion; insets represent magnified sections of the mitochondrion; arrows show cyclin B1; M: mitochondrion.

The observation that cyclin B1 translocates to mitochondria in irradiated cells was also supported by results obtained from electron microscopy (Figure 2). Consistent with the confocal microscopy images (Figure 1), cyclin B1 was found to be localized to the cytoplasm and nucleus in un-irradiated NHFs (Figure 2, control panel). However, NHFs irradiated with 6 Gy showed cyclin B1 protein translocating to the mitochondrion (Figure 2, 6 Gy panels).

**Figure 3:**
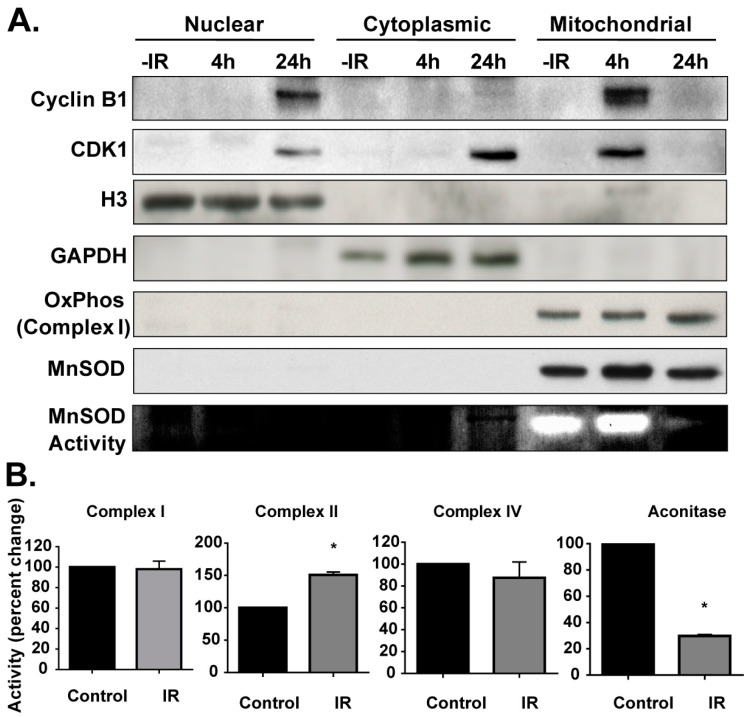
Cyclin B1 coordinates nuclear and mitochondria cross-talk. (**A**) Control (0 h) and 6 Gy irradiated NHFs were harvested at indicated times. Nuclear, cytoplasmic, and mitochondria fractions were isolated using the Qiagen cell fractionation kit, following the manufacturer’s supplied protocol. The purities of various fractions were determined by re-probing the blot with antibodies to histone H3 (nuclear), glyceraldehyde-3-phospahte dehydrogenase (GAPDH) (cytoplasm), oxidative Phosphorylation complex 1 39 kDa subunit (OXPHOS complex 1) and MnSOD (mitochondria). MnSOD activity (bottom panel) was measured by native gel electrophoresis. (**B**) Control (0 h) and 6 Gy irradiated NHFs were harvested at 24 h post-irradiation and mitochondria fractions were assayed for activities of complex I, II, IV and aconitase; asterisks indicate significant differences between control and irradiated cells (*n* = 3, *p* < 0.05).

Results obtained from the confocal and electron microscopy assays were further verified, using cell fractionation assays. NHFs, synchronized by contact inhibition (<2% S phase, measured by flow cytometric analysis of DNA content), were re-plated at a lower density and irradiated with 6 Gy at 18 h post-plating. Control and irradiated cells were harvested and used for cell fractionation, using the cell fractionation kit from Qiagen, following the manufacturer’s supplied protocol. Antibodies, specific to nuclear, cytoplasmic, and mitochondria, showed the purity of the cell fractionation (Figure 3A).

Consistent with the results presented in Figure 1 and Figure 2, results from the cell fractionation and immunoblotting assay showed cyclin B1 mitochondrial translocation at 4 h post-irradiation. Cyclin B1 protein levels in mitochondria were minimal at 24 h post-irradiation, while there was a significant accumulation of cyclin B1 in the nuclear fractions. The presence of cyclin-dependent kinase 1(CDK1) in the mitochondria suggests that cyclin B1 translocated with its associated kinase, CDK1. Cyclin B1 mitochondria translocation preceded a significant decrease in MnSOD activity. However, MnSOD protein levels showed only minimal changes, suggesting that the cyclin B1 translocation to the mitochondrion could directly or indirectly affect mitochondrial resident proteins, which could then affect mitochondria functions. To further examine if cyclin B1 translocation to mitochondria changes mito-protein functions, activities of four representative mitochondrial proteins were measured: complexes I, II and IV of the electron transport chain and aconitase of the tricarboxylic acid (TCA) cycle (Figure 3B). Cyclin B1 translocation to mitochondria did not change activities of complex I and complex IV, but the activity of complex II showed a 50% increase (Figure 3B). Aconitase activity was found to be significantly decreased by 70% compared to the control (Figure 3B). These results suggest that cyclin B1 translocation to mitochondria may significantly change the mito-proteome, thereby affecting mitochondrial functions.

### 3.2. Cyclin B1 Mitochondria and Nuclear Cross-Talk Was Associated with Oxidative Stress Induced Delay and Restart of the Cell Cycle

**Figure 4:**
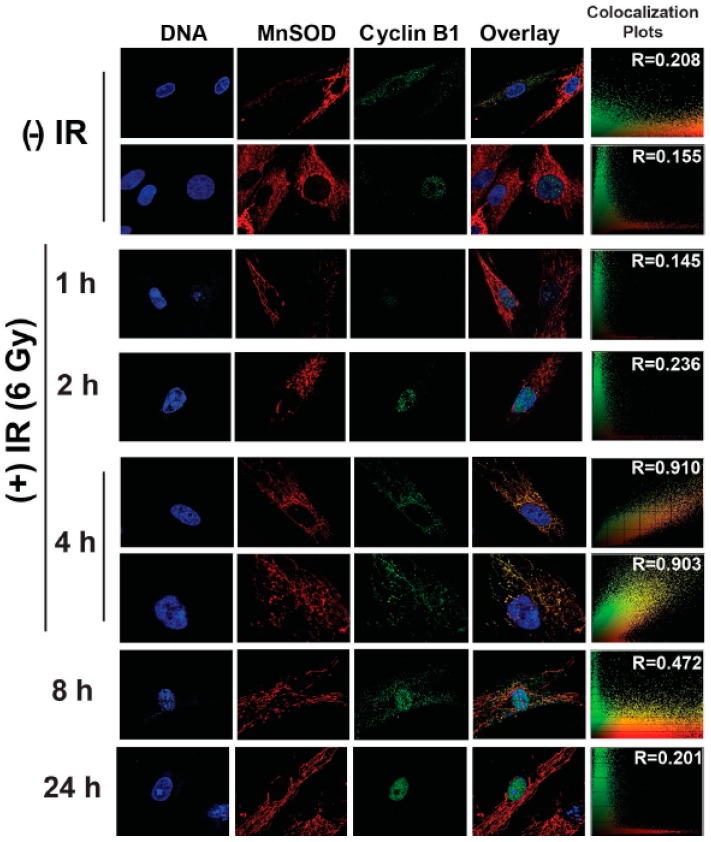
Cyclin B1 mitochondrial translocation following oxidative stress is a transient response. Synchronized NHFs were irradiated with 6 Gy and harvested at the indicated time post-irradiation, for immunostaining and microscopy assay, following the protocol described in the materials and methods section. Two separate fields are shown for the 4 h post-irradiation.

Cyclin B1’s mitochondrial translocation in irradiated cells appears to be a transient event. To further investigate this, synchronized NHFs representative of late S and G_2_ phases were irradiated with 6 Gy and assayed for microscopy at 1–24 h post-irradiation. Results presented in Figure 4 showed cyclin B1 translocated to the mitochondrion between 1 h and 4 h post-irradiation; its distributions were both cytoplasmic and nuclear at 8 h post-irradiation, and by 24 h post-irradiation cyclin B1 showed mostly nuclear accumulation.

**Figure 5:**
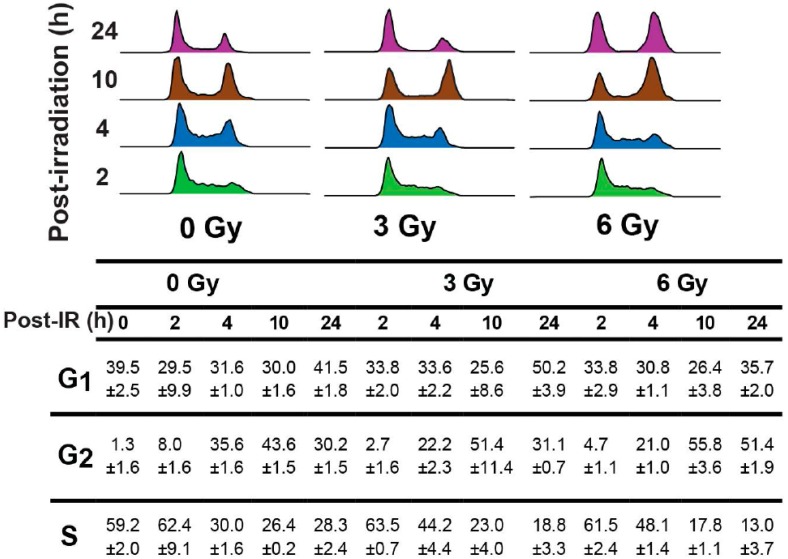
Cyclin B1 mitochondrial and nuclear translocations were associated with the delay and restart of the cell cycle. Confluent NHFs were re-plated and irradiated with 3 and 6 Gy at 18 h post-replating. Control and irradiated cells were harvested at indicated times for flow cytometry analysis of cell cycle phase distributions. A representative propidium iodide (PI)-histogram of DNA content is shown on the top panels. The percentage of cell cycle phases was calculated using the FlowJo software (FlowJo LLC, Ashland, OR, USA) and is expressed as mean ± standard deviation.

Cyclin B1’s mitochondrial translocation correlated with redistributions in the cell cycle phases (Figure 5). The percentage of G_2_ cells in 3 and 6 Gy irradiated NHFs at the time of irradiation was approximately 2–4%. The percentage of G_2_ cells increased to 20–50% between 2 h and 10 h post-irradiation, indicating a G_2_-delay. In 3 Gy irradiated cells, the percentage of G_2_ cells decreased to approximately 30% at 24 h post-irradiation, indicating a restart of the cell cycle.

### 3.3. Cyclin B1 Mitochondrial Translocation Perturbs Mitochondria Functions

**Figure 6:**
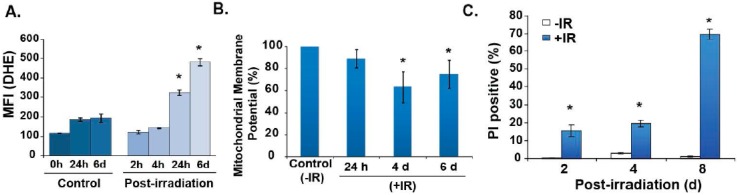
Cyclin B1 mitochondrial translocation precedes changes in mitochondria functions. NHFs, representative of late S and G_2_ phases, were irradiated with 6 Gy and harvested at indicated times for the measurement of mitochondria functions. (**A**) Flow cytometry measurements of DHE oxidation; results were normalized to time-matched un-irradiated controls and percent change, calculated relative to the 0 h time point. MFI: mean fluorescence intensity; * *n* = 3, *p* < 0.05. (**B**) Flow cytometry measurements of JC-1 fluorescence were used to determine changes in mitochondria trans-membrane potential. Results were calculated relative to the control. CCCP mitochondria un-coupler was used as a positive control for the assay (data not shown), * *n* = 3, *p* < 0.05. (**C**) Cell viability was assayed in live cells, using propidium iodide (PI) staining and flow cytometry. Cells were harvested by trypsinizing monolayer cultures, washed with cold PBS and PI was added prior to the flow cytometry assay. The percentage of PI positive cells (non-viable) was calculated using the FlowJo software. * *n* = 3, *p* < 0.05.

To determine if cyclin B1 mitochondrial translocation modifies mitochondria functions, NHFs, representative of late S and G_2_ phases, were irradiated with 6 Gy and harvested at indicated times, for measurements of mitochondria functions. Intracellular reactive oxygen species (ROS) levels were determined by flow cytometry measurements of dihydroethidium (DHE) oxidation. Initially, there was no significant difference in DHE fluorescence within 0–4 h post-irradiation (Figure 6A, left panel). The absence of any significant changes during 2–4 h post-irradiation compared to 0 h is comparable to our previously published results (2, 3, 6). DHE oxidation in 6 Gy irradiated NHFs increased approximately 3–5 fold at 24 h and 6 d post-irradiation. The initial increase in ROS levels at 24 h preceded the restart of the cell cycle (Figure 5), while the later increase in ROS levels at 6 d post-irradiation preceded the loss in mitochondria trans-membrane potential and cell viability (Figure 6B,C).

### 3.4. Mitochondria-Targeted Cyclin B1 Overexpression Mimics Many of the Cellular Properties Observed Following Oxidative Stress

**Figure 7:**
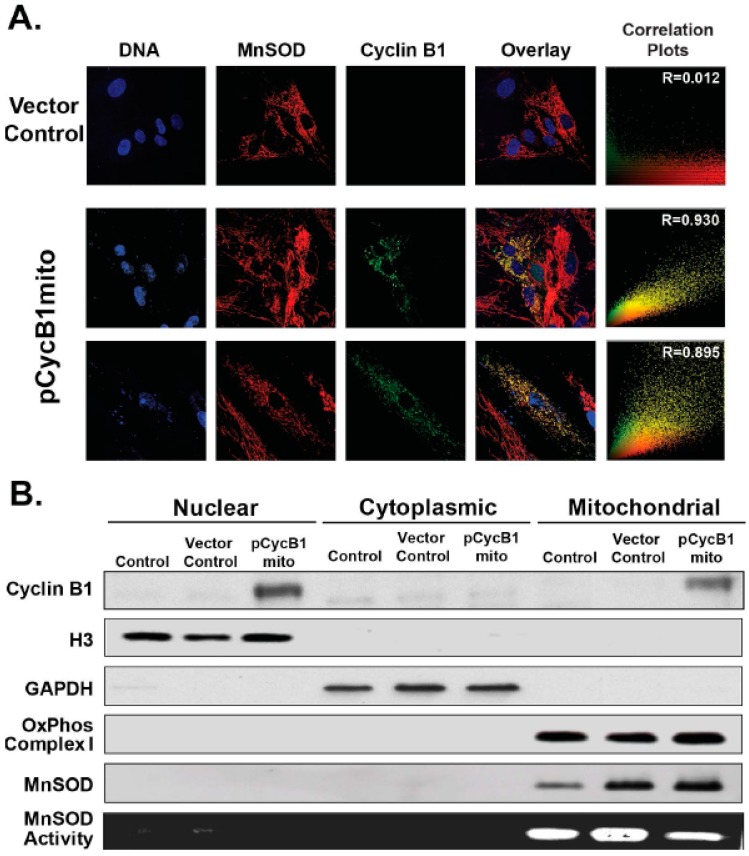
Mitochondrial overexpression of cyclin B1. Quiescent NHFs (1 × 10^6^) were transfected with 0.5 microgram of plasmid DNA and Arrest-In Transfection Reagent (Open Biosystem) following the manufacturer’s supplied protocol. Replicate dishes were transfected with pShooter GFP plasmid DNA and the transfection efficiency was calculated to be approximately 50%. (**A**) Control and plasmid transfected cells were harvested and transgene expression was verified by immunostaining and microscopy assay. Confocal microscopy, and calculation of correlation coefficient. (**B**) Control and plasmid transfected cells from a separate experiment were harvested for cell fractionation and western blotting assays.

To further determine if cyclin B1 mitochondrial translocation affects mitochondria functions, cyclin B1 was targeted for expression in the mitochondrion. The 1.5 kb human cyclin B1 cDNA, cloned into a pShooter vector (pCMV/myc/mito, Invitrogen), was used to transfect quiescent NHFs that do not express cyclin B1. Control and transfected cells were harvested for immunostaining and microscopy assay for transgene expression. The results (Figure 7A) showed MnSOD positive staining (red) in vector-control transfected quiescent NHFs without any cyclin B1 staining. However, quiescent NHFs transfected with mitochondria-targeted cyclin B1 showed positive staining (green) both in the nucleus and mitochondria, and mitochondrial staining of MnSOD (red). Overlay images of MnSOD (red) and cyclin B1 (green) showed that cyclin B1 was present in the mitochondrion (R = 0.9). These microscopy results were further verified from immunoblotting results obtained from a cell fractionation assay (Figure 7B). Mitochondria-targeted cyclin B1 overexpression showed its distributions both in the mitochondrion and nucleus. It is unclear if the mitochondria-localized cyclin B1 is transported to the nucleus once its mitochondria localization signal is cleaved off. Alternatively, the cytoplasmic retention signal/nuclear localization signal sequences in the endogenous cyclin B1 may also direct cyclin B1 to the nucleus under conditions of overexpression. It is interesting to note that the mitochondria-targeted overexpression of cyclin B1 was associated with a decrease in MnSOD activity (Figure 7B, bottom panel) without any corresponding changes in its protein levels (Figure 7B, 5th panel).

**Figure 8:**
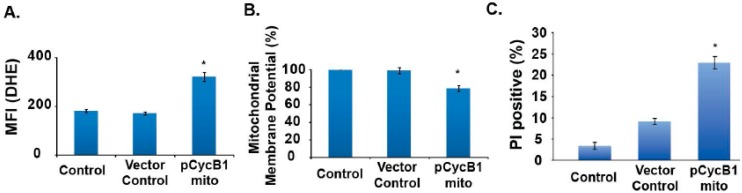
Mitochondria-targeted overexpression of cyclin B1 increased ROS levels, which was associated with a loss in mitochondria transmembrane potential, and cell viability. Quiescent NHFs were transfected following the protocol described in Figure 7. Control and transfected cells were harvested at 48 h post-transfection and used for flow cytometry measurements of (**A**) ROS levels, DHE oxidation, (**B**) mitochondrial transmembrane potential, JC-1 fluorescence, and (**C**) cell viability, PI staining of unfixed cells. *n* = 3, * *p* < 0.05.

To determine if mitochondria-targeted cyclin B1 expression alters mitochondrial functions, control and transfected cells were harvested and assayed for ROS level, mitochondria transmembrane potential, and cell viability. Mitochondria-targeted cyclin B1 expression increased DHE oxidation (~1.5-fold) compared to controls (Figure 8A). The increase in ROS levels correlated with a decrease (~20%) in mitochondria transmembrane potential (Figure 8B). Flow cytometry measurements of cell viability showed a ~25% loss in cell viability in mitochondria-targeted cyclin B1 overexpressing quiescent NHFs compared to controls (Figure 8C).

## 4. Discussion

Recent evidence, including our earlier research, has suggested that mitochondria-signaling could have a fundamental role in cellular proliferation, mitogenesis, inter-organelle communication, metabolic transitions, and physiological and environmental stresses [5,8,12,13,14,15]. Results from Figure 1, Figure 2 and Figure 3 clearly show cyclin B1 translocating to mitochondria after the radiation treatment, suggesting that cyclin B1 could integrate mitochondria function and cell proliferation in response to oxidative stress. Cyclin B1’s mitochondrial translocation could coordinate mitochondrial and nuclear functions during oxidative stress-induced G_2_ + M-delay and the subsequent restart of the stalled cell cycle.

Interestingly, cyclin B1 mitochondria translocation preceded a significant decrease in MnSOD activity (Figure 3 and Figure 7). However, MnSOD protein levels showed only minimal changes, suggesting that the cyclin B1 translocation to the mitochondrion could post-translationally modify mitochondria resident proteins. Affecting mitochondrial functions by post-translationally modifying the mito-proteome, could result in altered cellular activity, including cellular proliferation and viability. This was also supported by the differential changes in complex II and aconitase activity. Several recent reports have provided evidence for the mitochondrial translocation of protein kinases (protein kinase A, stress-activated protein kinase also called c-jun NH2-terminal kinase (JNK), P38 and extracellular receptor kinase/mitogen activate protein kinase (ERK/MAP), protein kinase anchoring protein (AKAP), Raf kinases, and SRCtyrosine kinase) [16,17]. Although the mitochondria targets of all these various kinases are not well defined, protein kinase A (PKA) phosphorylation stimulates mitochondrial translation and importation of MnSOD, a key nuclear encoded mitochondria matrix localized antioxidant enzyme that neutralizes mitochondria-generated superoxide [16]. Furthermore, the presence of CDK1 in mitochondria (Figure 3) suggests mitochondria localized cyclin B1/CDK1 could have kinase activity and MnSOD could be a substrate for cyclin B1/CDK1 kinase complex. This is consistent with two recent interesting reports showing that cyclin B1 and CDK1 translocate to the mitochondrion and post-translationally modify mito-proteins. In the first report, CDK1 translocated to the mitochondrion in cells treated with photodynamic therapy (PDT) [18]. Mitochondrial translocation of CDK1 correlated with Bcl-2 phosphorylation and PDT-induced cell death. In the second study, the authors showed that the cyclin B1/CDK1 kinase complex translocates to the mitochondria during the adaptive radiation response, and this translocation resulted in MnSOD phosphorylation at ser106 and an increase in its activity improving cellular survival [4]. These previous reports and results from this study further support the concept of a cross-talk between nuclear and mitochondria functions, which coordinates cellular responses to oxidative stress-response.

Our results show that cellular responses to oxidative stress are dependent on mitochondrial function, changes in the cellular redox environment and ROS levels (Figure 6 and Figure 8). The absence of any significant changes during 2–4 h post-irradiation compared to 0 h is comparable to our previously published results [19,20,21,22]. In our previous report, we used electron paramagnetic resonance (EPR) spectroscopy to measure the steady-state levels of superoxide in human oral squamous carcinoma cells irradiated with 6 Gy. These results showed no significant change in ROS levels in irradiated cells within hours of the radiation treatment [21]. Thus, results from our earlier and present studies indicate that the radiation-induced ROS generation, immediately after the treatment, is essentially similar to the ROS levels in un-irradiated control cells. However, cyclin B1’s mitochondrial translocation and subsequent post-translational modifications of the mito-proteome could change mitochondria functions that could perturb the steady-state levels of cellular ROS at a later stage. This “late ROS accumulation”, long after the radiation treatment, could be a significant factor determining cellular responses to radiation-induced oxidative stress. We have recently shown that the DHE oxidation increased approximately 2-fold in irradiated human glioma cells, 2–8 days post-irradiation [20]. This increase in ROS levels preceded the increase in the percentage of cells with sub G_1_-DNA content, indicating that the increase in ROS is not due to cell death, but rather is indicative of the activation of ROS-sensitive cellular processes leading to cell death. Because SOD overexpression suppressed late accumulation of ROS and SOD activity is well known to scavenge superoxide, our results also indicate that the major component of the radiation induced “late ROS accumulation” could be superoxide [20]. In a recent study, we showed that late ROS accumulation in radiation treated NHFs occurs after the activation of the cell cycle checkpoint pathways and precedes cell death [22]. These previous results are consistent and similar to the results we are seeing in this study with radiation treatment of NHFs (Figure 6 and Figure 8). DHE oxidation in irradiated NHFs increased approximately 3–5 fold at 24 h and 6 days post-irradiation. The increase in ROS levels preceded the loss in mitochondrial trans-membrane potential and cell viability (Figure 6 and Figure 8). These results suggest that the translocation of cyclin B1 to the mitochondrion and potential post-translational modification of the mito-proteome (e.g., MnSOD, complex II and aconitase) subsequently could alter ROS levels, mitochondria trans-membrane potential, and cell viability.

## 5. Conclusions

The results of this study and earlier evidence support the premise that cyclin B1’s mitochondrial translocation modifies the mito-proteome and activates a redox sensitive mitochondrial checkpoint, regulated by MnSOD (“mito-checkpoint”), initiating cell cycle delays and later, the restart of the stalled cell cycle. This cross-talk between “mito”- and “nuclear”-checkpoints is probably essential to avoid aberrant cell division, and protect the integrity of nuclear and mitochondria genome.

## Abbreviations

AKAP	A-kinase anchor proteins
BCl-2	B-cell lymphoma 2
CCCP	Carbonyl cyanide m-chlorophenyl hydrazone
CDK1	Cyclin-dependent kinase 1
CuZnSOD	Copper Zinc Superoxide Dismutase
DHE	Dihydroethidium
DMEM	Dulbecco’s modified Eagle’s medium
EPR	Electron paramagnetic resonance
ERK	Extracellular Signal-regulated Kinase
FBS	Fetal Bovine Serum
GAPDH	Glyceraldehyde-3-phospahte dehydrogenase
Gy	Gray
h	hour(s)
HBSS	Hanks buffer salt solution
IR	Ionizing radiation
JNK	c-Jun NH2-terminal kinase
MAP	Mitogen-activated protein
MCF10A	Human Mammary Epithelial cells
MFI	Mean fluorescence intensity
MnSOD	Manganese Superoxide Dismutase
NADH	Nicotinamide adenine dinucleotide
NBT	Nitroblue tetrazolium
NHF	Normal Human Skin Fibroblast
PBS	Phosphate Buffered Saline
PDT	Photodynamic therapy
PI	Propidium Iodide
PMT	photomultiplier
ROS	Reactive oxygen species
SDS-PAGE	Sodium dodecyl sulfate polyacrylamide gel electrophoresis
SRC	SRC proto-oncogene, non-receptor tyrosine kinase
TCA	Tricarboxylic acid cycle
